# Bioinformatics Analysis of Phylogeny and Transcription of TAA/YUC Auxin Biosynthetic Genes

**DOI:** 10.3390/ijms18081791

**Published:** 2017-08-18

**Authors:** Axel Poulet, Verena Kriechbaumer

**Affiliations:** 1 Department of Biological and Medical Sciences, Oxford Brookes University, Oxford OX3 0BP, UK; axel.poulet@emory.edu; 2 Department of Biostatistics and Bioinformatics, Rollins School of Public Health, Emory University, Atlanta, GA 30322, USA

**Keywords:** auxin, indole-3-acetic acid (IAA), endoplasmic reticulum (ER), transmembrane domain (TMD), phylogeny, RNAseq, YUCCA (YUC), tryptophan aminotransferase of arabidopsis (TAA), tryptophan aminotransferase related (TAR), gene duplication

## Abstract

Auxin is a main plant growth hormone crucial in a multitude of developmental processes in plants. Auxin biosynthesis via the tryptophan aminotransferase of arabidopsis (TAA)/YUCCA (YUC) route involving tryptophan aminotransferases and YUC flavin-dependent monooxygenases that produce the auxin indole-3-acetic acid (IAA) from tryptophan is currently the most researched auxin biosynthetic pathway. Previous data showed that, in maize and arabidopsis, TAA/YUC-dependent auxin biosynthesis can be detected in endoplasmic reticulum (ER) microsomal fractions, and a subset of auxin biosynthetic proteins are localized to the ER, mainly due to transmembrane domains (TMD). The phylogeny presented here for TAA/TAR (tryptophan aminotransferase related) and YUC proteins analyses phylogenetic groups as well as transmembrane domains for ER-membrane localisation. In addition, RNAseq datasets are analysed for transcript abundance of YUC and TAA/TAR proteins in *Arabidopsis thaliana*. We show that ER membrane localisation for TAA/YUC proteins involved in auxin biosynthesis is already present early on in the evolution of mosses and club mosses. ER membrane anchored YUC proteins can mainly be found in roots, while cytosolic proteins are more abundant in the shoot. The distribution between the different phylogenetic classes in root and shoot may well originate from gene duplications, and the phylogenetic groups detected also overlap with the biological function.

## 1. Introduction

Auxin is the major plant growth hormone and is involved in most developmental processes in plants. The multiplicity of auxin biosynthetic pathways that can be independently and differentially regulated result in a metabolic network which is capable of rapid end efficient dynamic changes. To understand the mechanism of local auxin action, it is crucial to determine the regulation of auxin biosynthesis. The currently most-researched pathway of auxin biosynthesis is the tryptophan aminotransferase of arabidopsis (TAA)/YUCCA (YUC) route involving tryptophan aminotransferases and YUC flavin-dependent mono-oxygenases to produce the auxin indole-3-acetic acid (IAA) from tryptophan.

The two-step pathway from tryptophan (Trp) to IAA, catalysed by the TAA proteins and the YUC flavin-containing mono-oxygenases, is the first identified complete auxin biosynthetic pathway, which is essential for almost all of the major developmental events in plants [1]. In the TAA/YUC-route, TAA/TAR (tryptophan aminotransferase related) enzymes convert Trp to indole-3-pyruvic acid (IPyA) which is then converted by YUC proteins to IAA [2]. YUC enzymes were first identified as key auxin biosynthesis enzymes as the overexpression of YUC in arabidopsis leads to auxin overproduction [3]. Importantly YUC enzymes were also identified via the first ever auxin biosynthetic mutants [4]. Inactivation of the first step in TAA/YUC-dependent auxin biosynthesis (TAA1 and its close homologues TAR1 and TAR2) leads to partial auxin deficiency and defects in several developmental processes [5]. With 11 *YUC* genes and three *TAA/TAR* genes in arabidopsis, this important pathway shows a high redundancy. Nonetheless, the disruption of multiple *YUC* genes causes dramatic developmental defects mainly in roots; these root phenotypes can be rescued by adding auxin to growth media [6].

### 1.1. Subcellular Location of Auxin Biosynthetic Genes

We have previously showed that, in maize [7] as well as in arabidopsis [8], auxin biosynthetic activity can be found in microsomal fractions, and a subset of auxin biosynthetic proteins show endoplasmic reticulum (ER)-localisation, mainly due to transmembrane domains (TMD). This could indicate a model of auxin function using ER membrane localisation and subcellular compartmentation for additional layers of regulation, which raises a question about the localisation of all the components of the arabidopsis TAA/YUC route.

Arabidopsis appears to use two separate sets of *YUC* genes for auxin biosynthesis in roots and shoots. YUC1, 2, 4 and 6 are the main YUC proteins in shoots, and YUC3, 5, 7, 8 and 9 are responsible for producing auxin in roots [5]. This is underlined by mutant studies: *yuc1/2/4/6* quadruple mutants have severe defects in vascular patterning and flower development but have no root defects, which is consistent with the expression patterns [6]. YUC3, 5, 7, 8 and 9 are expressed during root development and quintuple mutants of these five *YUC* genes (*yucQ* mutant) develop short and agravitropic root patterns [6]. The *yucQ* phenotypes are rescued by IAA addition or by expressing a *YUC* gene in *yucQ* roots but not by overexpression of a *YUC* gene in shoots [6]. This indicates that local auxin biosynthesis in roots is necessary for root development and that auxin transported from shoots is not sufficient. Interestingly, the shoot YUCs 1, 2, 4 and 6 are cytosolic, whereas YUCs located in roots (YUC5, 7, 8 and 9) are ER membrane-bound [8]. YUC3 is the only cytosolic YUC protein in the root. In contrast, arabidopsis seedlings appear to use the same set of *TAA*/*TAR* genes in both roots and shoots [5,9].

The ER is also interesting in terms of auxin transport, as recent data shows ER-specific PIN-FORMED (PIN) and PIN-LIKES (PILS) protein families of auxin transporters. Localization studies revealed that PIN5, PIN6, and PIN8 mainly localize to the ER [10,11,12,13,14].

As there is this apparent division between membrane and cytosolic proteins in roots and shoots, the evolution and the protein abundance of the different proteins is of interest.

We present here a phylogenetic analysis for TAA/TAR and YUC proteins that phylogenetic groups takes into account as well as transmembrane domains for ER-membrane localisation. Along with this, RNAseq datasets are mined to analyse the transcript abundance of YUC, TAA, and TAR proteins in *Arabidopsis thaliana*.

## 2. Results

### 2.1. Phylogenetic Analysis of YUC Proteins

**Figure 1:**
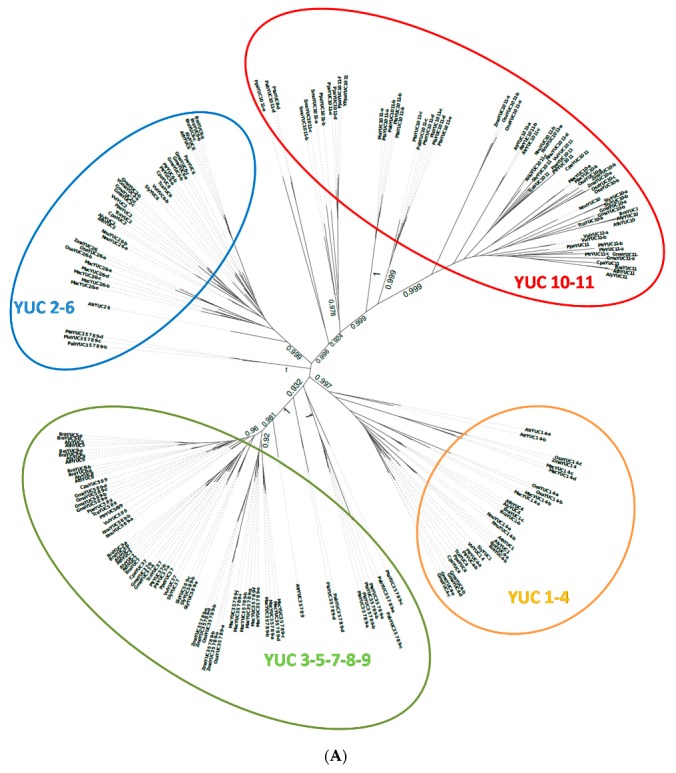

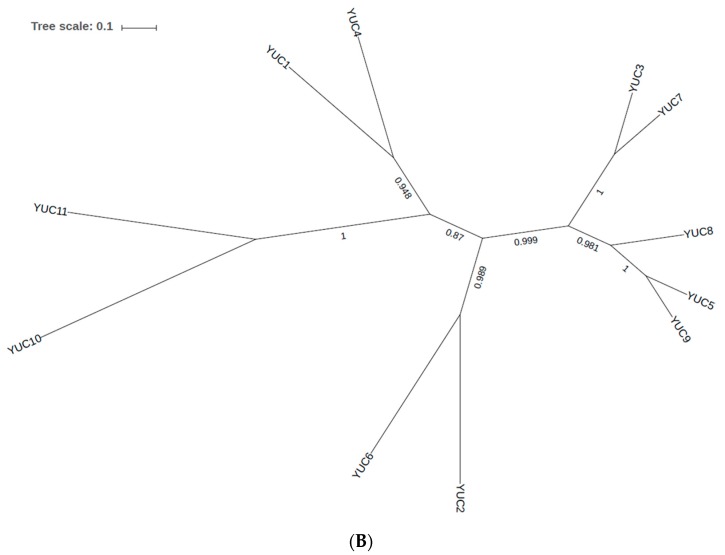
Phylogenetic tree of YUC proteins. Phylogenetic unrooted trees created using maximum likelihood methods on the proteins of interest resulting from an alignment are shown. Bootstrap values are given on the branches [16]. (**A**) Phylogenetic tree of all the homologues proteins detected for the 11 YUC proteins of arabidopsis. Bootstraps are only given in the branches, which split the different YUC clusters. Gene labels are constructed with the three letters from the species name (Table 1) and the gene name of the *Arabidopsis thaliana* homologues (Table S1); (**B**) Phylogenetic tree of the 11 YUC proteins detected in *A. thaliana*.

The eleven YUC proteins in *A. thaliana* can be grouped in four different groups (Figure 1). Five proteins comprise the first group: YUC3, YUC5, YUC7, YUC8, and YUC9. Here, two smaller subgroups can be defined, with YUC3 and YUC7 grouping together and YUC5, YUC8 and YUC9 also grouping together. The three other groups are YUC1–YUC4, YUC2–YUC6 and YUC10–YUC11.

During the homologous protein detection, 187 homologous YUC proteins were identified within all the species analysed (Table 1 and Table S1). The phylogenetic tree of these proteins (Figure 1A) shows the same topology to the tree for *A. thaliana* YUCs (AtYUCs) in Figure 1B. A comparison of these two trees in Figure 1 displays the same protein clusters: YUC10–YUC11, YUC1–YUC4, YUC2–YUC6 and YUC3–YUC7–YUC8–YUC5–YUC9. As in arabidopsis, ER-localised and cytosolic auxin biosynthetic proteins mainly group together. In this analysis, the prediction for TMD and protein domains was included in the analysis (InterProScan version 64.0, EMBL-EB, Hinxton, United Kingdom) [15]. This could give an indication of whether membrane localisation is a more ancient form—in evolutionary terms—or was added later on.

#### 2.1.1. Phylogenetic Analysis for Shoot-Localised YUC Proteins

For the shoot-localised YUC 1, 4, 2 and 6 proteins, 63 homologous proteins were detected (29 for YUC1–YUC4 group and 34 for YUC2–YUC6 group, Table S1). These proteins are only detected in the angiosperms (basal angiosperm, monocots and eudicots). YUC1 and YUC4 homologues are specific to brassicaceae; hence an event of gene duplication occurred specifically in brassicaceae (Figure S1) whereas YUC2–YUC6 gene duplication occurred after speciation between eudicots and monocots (Figure S2).

The majority of the proteins detected as homologous for YUC1–YUC4 and YUC2–YUC6 do not possess the predicted TMD features (Figures S1 and S2). For predicted membrane localisation, it is of interest that the earliest detected YUC1–YUC4 proteins in basal angiosperms both feature the predicted TMD (Figure S1). Later on, such predicted TMD can only be found in one out of eight monocot proteins (OsYUC1 4-b) and the two *Nelumbo nucifera* proteins, YUC1-a and YUC1-b. In the brassicaceae, TMD are predicted for BraYUC4-b and an alternate splice isoform of the arabidopsis YUC4 not annotated here [17]. Overall, this indicates that membrane localisation for the YUC1/YUC4 group was mostly lost over time.

The ancestral basal angiosperms show no indication of TMD in YUC2–YUC6 analysis and only a few proteins in monocots (banana MacYUC2 6-d), eudicots (cocoa tree TcaYUC2, soybean GmaYUC6-b, papaya CpaYUC6) and brassicaceae (field mustard BraYUC6-a) feature predicted TMD (Figure S2).

#### 2.1.2. Phylogenetic Analysis for Root-Localised YUC Proteins

Sixty-five homologues were found in the YUC3–YUC7–YUC8–YUC5–YUC9 group; these proteins occur in gymnosperms and angiosperms, with no homologues detected in moss and club moss (Table S1). The majority of these proteins possess a predicted TMD on the N-terminus or in the middle of the protein, with the flavin-dependent monooxygenase domain constituting the rest of the protein.

**Figure 2:**
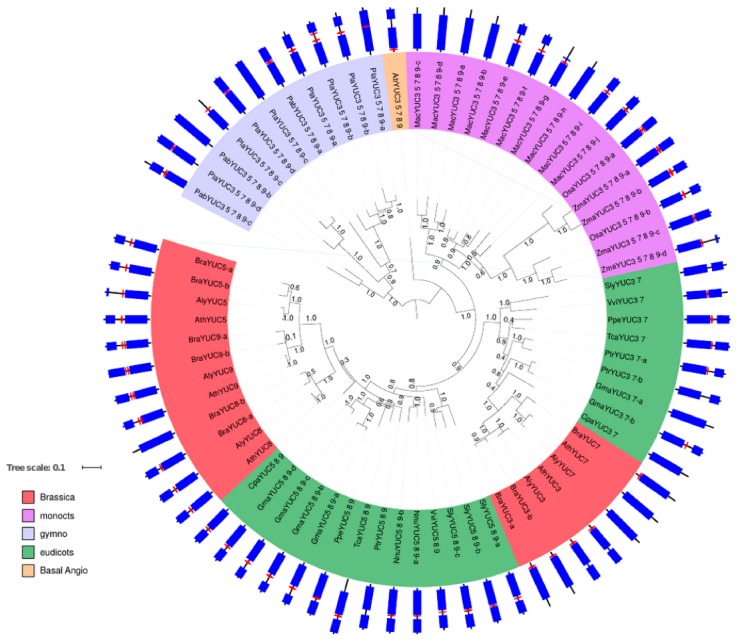
Phylogenetic tree of YUC3, YUC7, YUC8, YUC5 and YUC9 homologues proteins. A fast-tree using the maximum likelihood method [16] was applied on YUC3 YUC7 YUC8 YUC5 and YUC9 protein homologues constructed from an alignment using the MUSCLE (MUltiple Sequence Comparison by Log-Expectation) alignment tool with default parameters [18]. Bootstrap values are given on the branches. Gene labels are constructed with the three letters from the species name (Table 1) and the gene name of the *A. thaliana* homologues (Table S1). The color of the label shows the lineage of the plant. The corresponding protein domains (circle periphery) are obtained using InterProScan [15], red hexagons represent the TMD in the protein if applicable, and blue rectangles indicate the flavin-dependent monooxygenase domain typical for YUC proteins.

One homologue of the YUC3–7–8–5–9 group was detected in basal angiosperms; eudicot homologues form two clusters, one for the YUC3–YUC7 homologues and the other one for YUC5–YUC8–YUC9. This suggests that YUC3–YUC7 and YUC5–YUC8–YUC9 were duplicated after the speciation between eudicots and monocots (Figure 2). The duplication of YUC3–YUC7 as well as the two gene duplications for YUC8 and YUC5–YUC9 are specific to brassicaceae. This group of proteins goes back to gymnosperms, and most proteins in this group feature a predicted TMD. In gymnosperms, only loblolly pine (PtaYUC3 5 7 8 9-c) and Norway spruce (PabYUC3 5 7 8 9-b) do not feature such a membrane anchor, but this is complemented by three or two other proteins, respectively, in *Picea taeda* and *P. abies*, that feature a predicted TMD. In the basal angiosperms, only amborella codes for a YUC3-5-7-8 protein (AtrYUC3 5 7 8) which also shows a TMD prediction. In monocots, all proteins in rice and maize feature TMD. In banana (*Musa acuminata*), with the largest number of proteins from the YUC3–7–8–5–9 group, five out of ten feature a predicted TMD. In eudicots, all but one protein in the YUC5–YUC8–YUC9 group feature predicted TMD, with poplar PtrYUC5-8-9 being the exception. The eudicot YUC3–YUC7 group also mainly features predicted TMD (*Vitis vinifera*, *Prunus persica*, *Theobroma cacao*) or at least one of two proteins contain sequences indicative of a TMD (*P. trichocarpa*, *Glycine max*). Tomato (*Solanum lycopersicum*) and papaya (*Carica papaya)* YUC3–YUC7 proteins do not feature TMD. In brassicaceae, only *A. thaliana* and *A. lyrata* YUC7 as well as one of the *Brassica rapa* YUC8 proteins (BraYUC8-b) do not feature a TMD indication.

Overall, this is of interest as the YUC3–YUC7–YUC8–YUC5–YUC9 group is split in two groups from eudicots onwards: YUC3–YUC7 shows more of a tendency to cytosolic proteins and YUC5–YUC8–YUC9 to membrane-localised proteins. Throughout evolution, the proteins in the YUC5–YUC8–YUC9 group seem more consistent in featuring one or several predicted TMD than the YUC3–YUC7 group. This agrees with the arabidopsis data, where YUC5, 8, and 9 feature TMD but YUC7 does not, and YUC3 has a predicted TMD but still shows cytosolic localisation [8].

#### 2.1.3. Phylogenetic Analysis for YUC Proteins Localised in Flowers and Siliques

**Figure 3:**
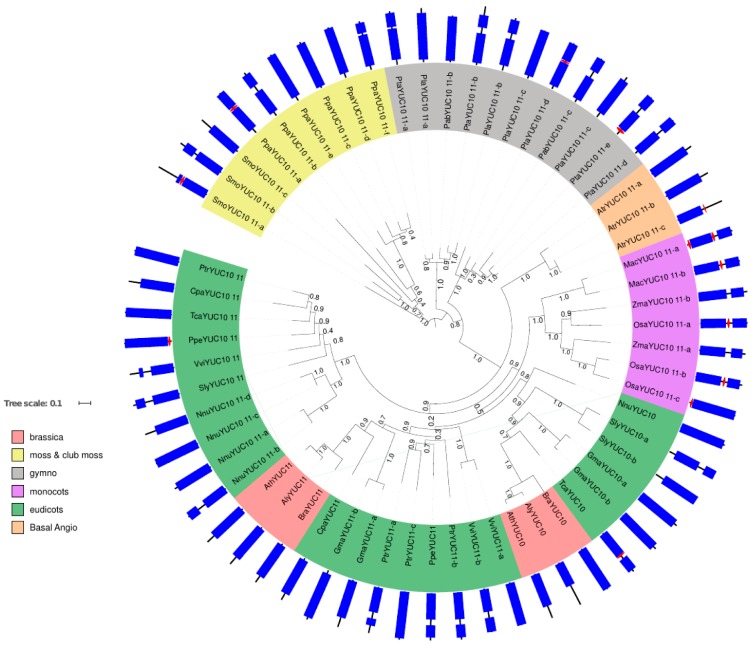
Phylogenetic tree of YUC10 and YUC11 homologous proteins. The tree is made using fast-tree with the maximum likelihood method [16] on YUC10 and YUC11 protein homologues constructed from an alignment using the MUSCLE alignment tool with default parameters [18]. Bootstrap values are indicated on the tree branches. Gene labels are constructed with the three letters from the species name (Table 1) and the gene name of the *A. thaliana* homologues (Table S1). The colour of the label shows the lineage of the plant. The corresponding protein domains (circle periphery) are obtained using InterProScan [15], red hexagons represent the TMD in the protein if applicable and blue rectangles indicate the flavin-dependent monooxygenase domain typical for YUC proteins.

In the YUC10–11 cluster, 59 proteins were found in all the species analysed. Therefore, these genes seem to be the most conserved in the YUC family and can be traced back to mosses and club mosses. In this group, the gene duplication between YUC10 and YUC11 occurred after the speciation between monocot and eudicots (Figure 3). The YUC10–YUC11 group features fewer TMD; e.g., in mosses and club mosses, only two proteins feature a predicted TMD. In gymnosperms, two *Pinus taeda* proteins (PtaYUC10 11-d and -e) and in basal angiosperms one amborella protein (AtrYUC10 11-c) potentially feature a TMD. Interestingly, in monocots, all banana and rice YUC10–YUC11 proteins have a TMD prediction but none of the maize proteins. Peach PpeYUC10 11 and cocoa TcaYUC10 11 show TMD predictions in the eudicots. None of the YUC10–YUC11 proteins in the brassicaceae analysed (*B. rapa*, *A. thaliana* and *A. lyrata*) show any TMD predictions (Figure 3).

In arabidopsis, AtYUC10 and AtYUC11 are suggested to have overlapping functions with At*YUC1* and At*YUC4* during embryogenesis [4]. All these proteins are cytosolic [8] except for the AtYUC4.2 splice variant which is flower specific [17]. Despite being seemingly the most ancient YUC isoforms, YUC10 and YUC11 seem to have adapted a specific role in embryogenesis in *A. thaliana* [4]. This is also represented in the RNAseq data for these genes below.

### 2.2. Phylogenetic Analysis of TAR and TAA Proteins

**Figure 4:**
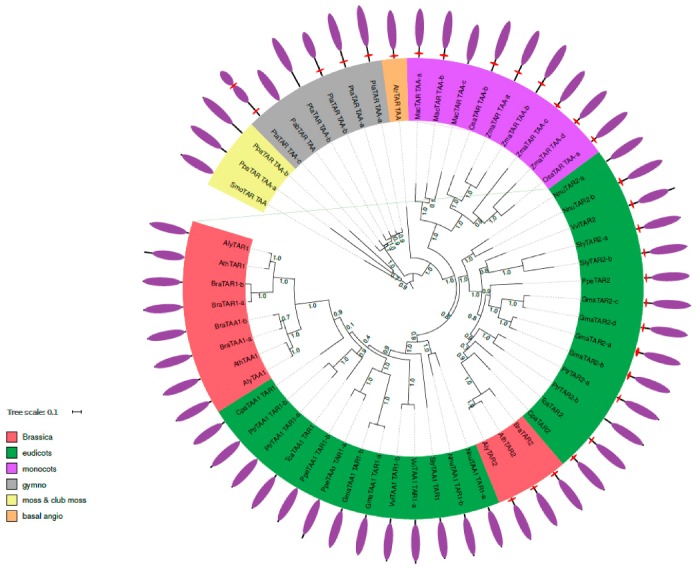
Phylogenetic tree of TAR1, TAR2 and TAA1 homologous proteins. Fast-tree using the maximum likelihood method [16] was used to create the phylogenetic tree for TAR1, TAR2 and TAA1 protein homologues constructed from MUSCLE alignment [18]. Bootstrap values are indicated on the branches of the phylogenetic tree. Gene labels are constructed with the three letters from the species name (Table 1) and the gene name of the *A. thaliana* homologue (Table S1). The colour of the label shows the lineage of the plant. The corresponding protein domains (circle periphery) are obtained using InterProScan [15], red hexagons represent the TMD in the protein if applicable and purple ellipses represent tryptophan aminotransferase domain.

Three *A. thaliana* proteins were used as references to perform the phylogenetic analysis, or TAA/TAR proteins: AtTAR1, AtTAR2, and AtTAA1 (Table 2). These three proteins possess a tryptophan aminotransferase domain, but only TAR2 features a predicted TMD in the N-terminal region. Due to the similarity in the homologues, a screen for the three proteins only one phylogenetic tree was created. The search with BLASTp and HMMER resulted in 57 proteins detected in all the species analysed (Table S2). The duplication of TAR1/TAA/TAR2 occurred during the speciation between monocots and eudicots. The eudicots clearly split into two groups: TAA1/TAR1 proteins featuring no predicted TMD and the TAR2 group (Figure 4). In these groups, most proteins indicate TMD, with the exceptions being one of the two lotus proteins NnuTAR2-a and one of two poplar proteins PtrTAR2-b. In brassicaceae, this division continues, but here all proteins in the TAR2 branch feature a predicted TMD and the cytosolic proteins are now divided into TAA1 and TAR1 proteins (Figure 4).

Following this duplication, TAR1 and TAA1 proteins show a high similarity. Both proteins are only found together in brassicaceae, so the duplication of these proteins occurred during brassicaceae speciation.

Regarding subcellular localisation throughout evolution for TAA/TAR proteins, the precursor proteins in mosses and club mosses are predicted to locate in the cytosol. In the gymnosperms, both *Pinus lambertiana* TAA/TAR proteins show TMD predictions, and two out of three *Pinus taeda* proteins do. Only *Picea abies* PabTAR TAA does not feature such a motif. In addition, the basal angiosperm amborella features a predicted TMD.

**Figure 5:**
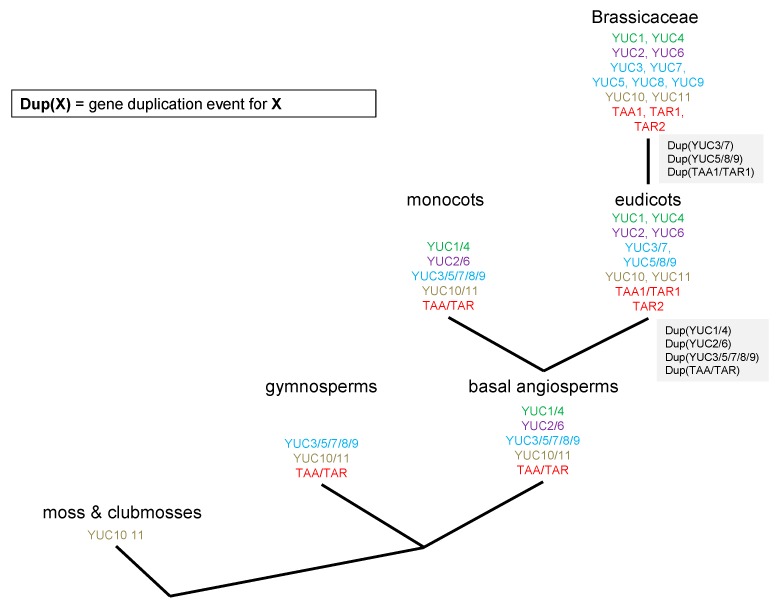
Evolutionary development of auxin biosynthetic enzymes in the TAA/YUC pathway. Nodes show an event of speciation, which is correlated with the appearance or the duplication of *YUC* or *TAR*/*YUC* genes. The name gene colors indicate the different “gene families”.

All in all, we are proposing that the phylogenetic groups detected also overlap with the biological function. YUC10–11 ancestry proteins are present in all species analysed, and therefore seem to be the oldest and potentially original proteins (Figure 5). During species diversification, their role seems to have become more specialised. In arabidopsis, AtYUC10 and AtYUC11 are suggested to have overlapping functions with At*YUC1* and At*YUC4* during embryogenesis [4]. A transcript for YUC10 is only detectable in flowers and siliques, and YUC11 is mostly transcribed in flowers (see RNAseq data below). In the YUC10–11 ancestral proteins, we have a mix of proteins with and without TMD predictions, with a lot of the monocot proteins featuring a predicted TMD, only two in eudicots and none in brassicaceae. YUC1–4 proteins occur for the first time in basal angiosperms, where they feature a TMD, whereas only one or two, respectively, TMD featuring YUC1/4 protein are detected in monocots and two representatives of the eudicots.

The next YUC group to develop is suggested to be YUC3–5–7–8–9, with over 95% of the proteins featuring predicted TMD; YUC3–5–7–8–9 then splits into two groups in eudicots, YUC3–YUC7 and YUC5–8–9 (Figure 5). Still, most of the proteins derived from these groups contain predicted TMD with YUC7 proteins being more likely not to carry a TMD.

Original TAA/TAR proteins do not feature predicted TMD; the first TMD prediction can be detected in gymnosperms and basal angiosperms. Interestingly, similar to YUC1 and YUC4 proteins, TAA and TAR proteins do not divide into two groups in monocots but do in eudicots. However, whereas YUC1 and YUC4 proteins mainly do not show TMD predictions in both proteins, the TAA/TAR groups splits into TAA and TAR1 proteins without, and, in the case of TAR proteins, mainly with, a TMD.

In this context, it is of interest that TAR1 and TAR2 single mutant displays no visible phenotype and TAA1 show altered responses to shade and a weak ethylene insensitivity that can be rescued by IAA but no developmental defects under normal growth conditions [5]. The TAA1/TAR2 double mutants have defects in their vascular and floral development and are sterile [5]. Triple mutants (TAA1/TAR1/TAR2) lack a primary root and visible vasculature in their cotyledons, have a reduced hypocotyl, and are more likely to develop a single cotyledon than wild-type embryos [5]. Other than YUC proteins that show defects in root or shoot, respectively, depending on whether the mutations are in ER-localised or cytosolic YUC proteins [6], TAA and TAR proteins seem to have overlapping functions in root and shoot tissue, which is also reflected in their RNAseq profile and will be discussed in the next paragraph.

### 2.3. RNAseq Analysis of Auxin Biosynthetic Enzymes in the TAA/YUC Pathway

In arabidopsis, auxin biosynthetic activity can be found associated with both cytosolic as well as ER-membrane fractions [8], and a subset of auxin biosynthetic proteins show ER-localisation, whereas other enzymes localize in the cytosol [8]. Mutant studies suggest YUC1, 2, 4 and 6 to be the main YUCs in shoots and YUC3, 5, 7, 8 and 9 in roots [6].

**Figure 6:**
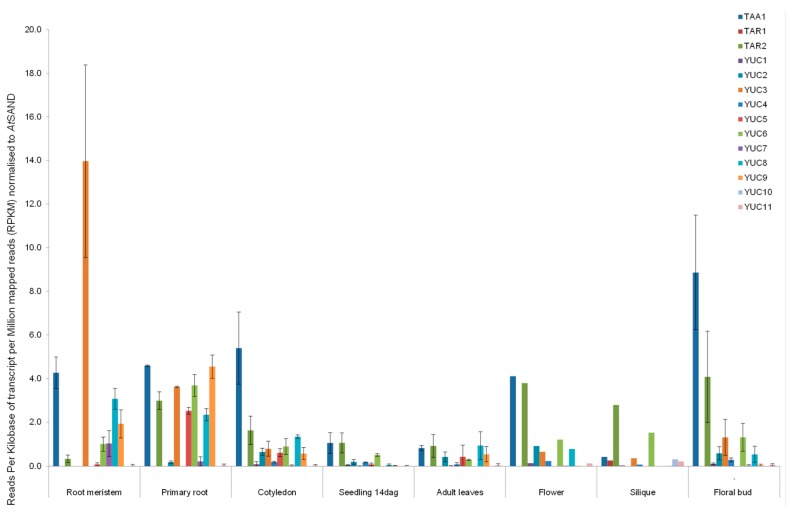
Tissue-specific distribution of TAA/TAR and YUC transcripts.

To analyse the tissue distribution of auxin biosynthetic enzymes, an RNAseq analysis for TAA1, TAR1, TAR2 as well as the 11 YUC transcripts was carried out (Figure 6 and Tables S1–S3). Datasets were obtained for the following tissues: root meristems, primary roots, cotyledons, 14-day-old seedlings, adult leaves, flowers, siliques, and floral buds. The data available on NCBI (https://www.ncbi.nlm.nih.gov/geo) were mapped against The Arabidopsis Information Resource (TAIR) database, and then the reads per kilobase of transcript per million mapped reads (RPKM) was computed for all genes of interest and normalised (see Material and Methods).

It is interesting to note that, for the first step of auxin biosynthesis, TAA1 is far more predominant than TAR2. Only in siliques can we find higher transcript levels for TAR2 than for TAA1 and approximately equal levels in flowers, as well as in seedlings and adult leaves. TAR1 is only present in flowers and siliques at very low transcript levels (Figure 6).

For the YUC genes and the second step in YUC-dependent auxin biosynthesis, YUC3 appears to be the predominant transcript in the root meristem and primary root. YUC3 is an interesting protein, as phylogenetically it groups with the ER-membrane bound YUC proteins and shows predictions for a TMD but shows cytosolic localization [8]. The ER-localised YUCs (YUC5, 7, 8, and 9) have their highest transcript levels in root meristem and primary root tissue. YUC5, 8 and 9 have a high level of amino acid identity (~75%) and are most likely the result of gene duplications occurring between the evolution of the eudicots and brassicaceae. YUC5 is mainly transcribed in primary roots. YUC8 transcript levels decrease from rot meristem over primary root to cotyledons, whereas YUC9 is at its highest level in primary root tissue. YUC7 is mainly transcribed in the root meristem (Figure 6).

In the cytosolic group, YUC1—probably the most studied YUC enzyme—and YUC4 are not detected in root meristem and primary root and are transcribed at very low levels otherwise, with a slight increase in flowers and floral buds. YUC6 is transcribed throughout the tissues analysed, with slightly higher transcription levels in primary roots and lower levels in adult leaves. YUC10 is only detectable in flowers and siliques at very low levels; the last cytosolic protein YUC11 is transcribed throughout at low levels with a slight maximum in flowers.

RNA sequencing transcript analysis was carried out for TAA1, TAR1, TAR2 and YUC1, 2, 3, 4, 5, 6, 7, 8, 9, 10, 11 in root meristem, primary root, cotyledon, seedling 14 days after germination, adult leaves, flower, silique and floral bud tissue (Table 3 and see Material and Methods section). Data is given as RPKM (reads per kilobase of transcript per million mapped reads) and was normalised to the reference gene *AtSAND* (At2g28390).

**Figure 7:**
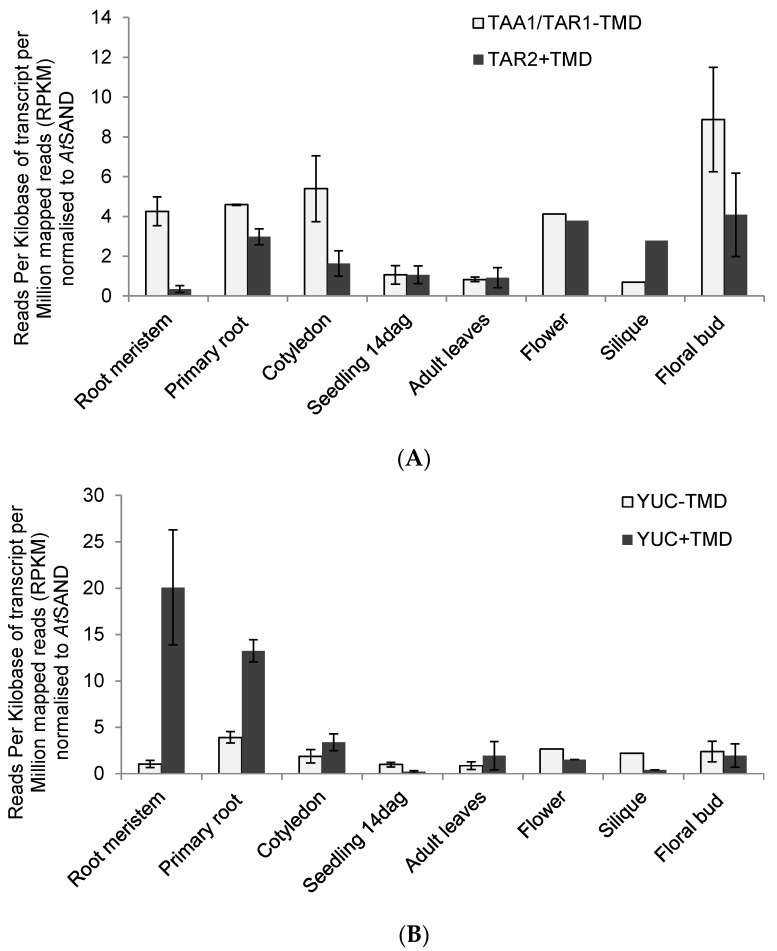
Tissue-specific distribution of TAA/TAR and YUC transcripts with and without predicted TMD. (**A**) RNA sequencing transcript analysis for TAA/TAR proteins without TMD (TAA1, TAR1) and with TMD prediction (TAR2) in root meristem, primary root, cotyledon, seedling 14 dag, adult leaves, flower, silique and floral bud tissue (Table 3 and see Material and Methods section). Data is given as RPKM (reads per kilobase of transcript per million mapped reads) and was normalised to the reference gene *AtSAND* (At2g28390); (**B**) RNAseq transcript analysis for YUC proteins without TMD (YUC1, 2, 4, 6, 10, 11) and with TMD prediction (YUC3, 5, 7, 8, 9) in root meristem, primary root, cotyledon, seedling 14 dag, adult leaves, flower, silique and floral bud tissue (Table 3 and see Material and Methods section) Data is given as RPKM (reads per kilobase of transcript per million mapped reads) and was normalised to the reference gene *AtSAND* (At2g28390).

To look into the distribution of proteins with and without the predicted TMD in the different tissues, and especially in terms of the mutant data [6] in root and shoot tissues, transcript levels for YUC1, 2, 4, 6, 10, and 11 and transcript levels for YUC3, 5, 7, 8, and 9 were compiled for each tissue (Figure 7).

For TAA/TAR, the cytosolic TAA1 and TAR1 which do not feature a predicted TMD show equal or higher transcript levels than the ER-membrane bound TAR2 with a predicted TMD, with the exception of silique tissue, where TAR2 is the main transcript (Figure 7A). For the YUC genes, the cytosolic YUCs are the main transcripts in seedlings, flower, silique and flower bud (Figure 7B). The main transcripts in root meristem and primary root are indeed the ER-membrane bound YUCs, together with YUC3 (Figure 7B). YUC3 does feature a predicted TMD but has not shown ER-localisation in plants [8]. YUC3 indeed accounts for the majority of transcript in root meristem tissue (Figure 6). These findings align very well with mutant data: YUC1–2–4–6 quadruple mutants have severe defects in vascular patterning and flower development but have no root defects, which is consistent with the expression patterns [6]. Quintuple mutants of YUC3, 5, 7, 8 and 9 (*yucQ* mutant) develop short and agravitropic roots [6]. The *yucQ* phenotypes are rescued by IAA addition or by expressing a YUC gene in *yucQ* roots but not by overexpression of a YUC gene in shoots [6]. The root phenotypes of the *yucQ* mutant (*yuc*3/5/7/8/9) can be rescued by YUC overexpression in the mutant roots but not in shoots [6], highlighting the necessity of local auxin biosynthesis. Furthermore, the phylogenetic class of YUC3, 5, 7, 8, and 9 seems to be mainly involved in auxin biosynthesis in the roots, while the class containing the cytosolic proteins YUC1, 2, 4, 6, 10, and 11 might be more important for auxin biosynthesis in shoots, with YUC10 and 11 only present in flowers and siliques.

## 3. Material and Methods

### 3.1. Homologous Protein Detection

For all proteins of interest, a basic local alignment search tool (BLASTp, NCBI, Bethesda, MD, USA) was used with default parameters as well as the hidden Markov model-based sequence alignment tool (HMMER, EMBL-EB, Hinxton, United Kingdom, http//hmmer.org). Best hits were retained and used for phylogenetic analysis [19]. The proteome of each species was used as reference for the two analyses (Table 1), and the YUC, TAR and TAA proteins of Arabidopsis were used as queries (Table 2). Analysis results are shown in Table S1 for YUC proteins and Table S2 for TAR/TAA proteins. InterProScan (EMBL-EB, Hinxton, UK) [15] was applied for domain predictions (TMD, flavin-dependent monooxygenase domains, tryptophan aminotransferase domains). InterProScan (EMBL-EB, Hinxton, UK) is a platform that integrates multiple and diverse databases for the domain prediction allowing for a comparative but integrated approach whilst reducing redundancy and capitalizing on the individual strengths of the different databases. Reciprocal BLASTp (NCBI, Bethesda, MD, USA) and domain analysis with InterProScan (EMBL-EB, Hinxton, UK) [15] were used to verify the relevance of all identified orthologues. The protein data used in this analysis were downloaded from three different databases: plaza genomic (http://bioinformatics.psb.ugent.be/plaza), phytozome (https://phytozome.jgi.doe.gov/pz/portal.html) and congenie (http://congenie.org/); nomenclature was used according to these databases.

### 3.2. Phylogenetic Reconstruction

Selected sequences were first aligned with MUSCLE (EMBL-EB, Hinxton, UK), a multiple sequence alignment tool [18], using default parameters. For the construction of the phylogenetic tree Fast-Tree was applied using the default parameters [16]. Fast-Tree infers approximately-maximum-likelihood phylogenetic trees from alignments. Finally, phylogenetic trees were drawn using the Interactive Tree Of Life ITOL (EMBL, Heidelberg, Germany) [20]. The protein domains shown in the different phylogenetic clades are detected with INTERPROSCAN (EMBL-EB, Hinxton, UK) [15] 

### 3.3. RNA Sequencing Data and Analysis

Data used for the RNAseq analysis was obtained from the NCBI (http://www.ncbi.nlm.nih.gov/geo/browse/). Five different tissues (leaves, roots, flowers, flower buds, and seeds/siliques) as well as total seedling were chosen for the analysis of the expression patterns of the genes of interest in *A. thaliana* (Table 3). Reads from RNAseq libraries were mapped onto the candidate gene sequences allowing no mismatches using TOPHAT v 2.0.14 (University of Maryland, MD, USA) [21] with standard settings and maximum of multihits set at 1, minimum intron length set at 15 bp, and maximum intron length set as 6000 bp. Reads were added together for each gene using HTseq-count with the overlap resolution mode set as intersection-non empty and with no strand-specific protocol [22]. Transcription levels in reads per kilobase of transcript per million mapped reads (RPKM) were normalised to *AtSAND* (At2g28390). This gene was chosen due to its constant gene expression levels across different tissues at developmental stages in *A. thaliana* [23].

### 3.4. Confocal Imaging of Arabidopsis Seedlings

*A. thaliana* seeds expressing GFP-HDEL in a stable manner were sterilized with 10% (*v*/*v*) bleach and then rinsed in sterile distilled, deionized water. The seeds were plated in petri dishes on Murashige and Skoog medium with 1% (*w*/*v*) sucrose, solidified with 1.2% (*w*/*v*) phytoagar, and grown in 16-h photoperiods at 22 °C for 7 days. Images were taken using a Zeiss 880 laser scanning confocal microscope (Zeiss, Oberkochen, Germany) with × 100/1.46 NA oil immersion objective. For imaging of the GFP samples were excited using the 488 laser line with detection in the 494- to 513-nm range. Images were edited using the ZEN image browser (Zeiss, Oberkochen, Germany).

## 4. Conclusions

**Figure 8:**
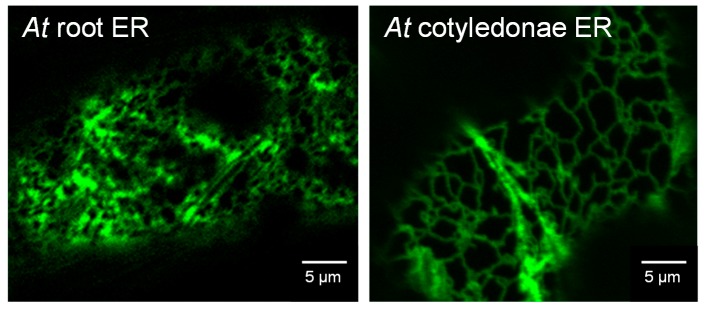
Structure of the endoplasmic reticulum (ER) in arabidopsis root and cotyledonae. Confocal images of ER in arabidopsis root and cotyledonae seven days after germination. Arabidopsis transformed in a stable manner, with the ER lumenal marker GFP-HDEL (green fluorescent protein fused to an ER retention signal consisting of the four amino acids Histidine-Aspartic acid-Glutamine-Leucine) used to visualize the ER.

We hypothesize that membrane anchoring via TMD to the endoplasmic reticulum in TAA/YUC auxin biosynthesis could already be found early on in evolution in mosses and club mosses. The distribution between the different phylogenetic classes in root and shoot may well originate from gene duplications. The original YUC10–11 proteins then adapted more specialized functions in flowers and siliques, together with cytosolic YUC1–YUC4 proteins. The phylogenetic group of YUC5,8,9 features predicted TMD throughout evolution and in arabidopsis can mainly be found and is active in root tissue. This might be linked to the high abundance of ER in arabidopsis roots compared to leaf tissue (Figure 8) and a difference in network structure as well as functional requirements of this tissue such as enhanced secretion. ER-linked auxin biosynthesis might therefore be important for a variety of plant species throughout evolution. So far, this has been tested in maize and arabidopsis. We have shown that, for maize coleoptiles and primary root [7] and arabidopsis whole seedlings [8], about a quarter of auxin biosynthetic activity can be found in microsomal fractions containing ER membranes. In the light of this phylogenetic and transcription data, it would therefore be of great interest to test the contribution of ER-localised and cytosolic auxin biosynthesis in different species throughout evolution and link it to the abundance of the corresponding proteins, with and without TMD. Narrowing down such analysis to different tissues, e.g., root and shoot in arabidopsis, also has the potential to reveal a difference in regulation of TAA/YUC auxin biosynthesis, which might be dependent on tissue function and subcellular structures etc.

For arabidopsis and the transcriptional data, it will be of great interest to further elucidate the role and contribution of YUC3 to auxin biosynthesis due to the high abundance of the transcript especially in the root meristem. It is also interesting that enzymes in the two steps of TAA/YUC-dependent auxin biosynthesis display differences in the tissue distribution between proteins with and without TMD: for the first step, TAA1 and TAR2 are the main forms, with the cytosolic TAA1 being more predominant than the ER-localised TAR2. TAR2 shows higher transcript levels than TAA1 only in siliques. In contrast to that, YUC proteins with predicted TMD show higher transcript levels in root tissues whereas proteins without predicted TMD seem to be higher transcribed in leaf and flower tissues (Figure 7). Enzymatic steps in one pathway should be more efficient if the enzymes are in proximity, or at least in the same subcellular compartment, if transport of intermediates is to be avoided. It is therefore curious that, for the first step in the biosynthesis, a cytosolic TAA1 is predominant, whereas for the second step, the YUC proteins show tissue specificity between TMD and non-TMD proteins. This could suggest protein–protein interactions and metabolon formation as suggested before for auxin biosynthesis [8] but detailed proteomics and localisation analysis including membrane topology are necessary to shed light on this matter.

All in all data, derived from phylogenetic analysis and arabidopsis transcription data fits well with mutant studies [6], indicating a subgroup of YUC proteins that are localized to the ER and seem predominant in root tissues. Already in the simple basal angiosperm, such as amborella, which underwent only few whole genome duplications, TAA/YUC-dependent auxin biosynthesis is already complex and displays differentiation in ER-membrane bound and cytosolic proteins. This could also indicate the conservation of differences for auxin biosynthesis in root and leaf since the basal angiosperms.

## Figures and Tables

**Table 1:** Species for the phylogenetic analysis of the TAA/YUC auxin biosynthetic proteins. Species abbreviation are used in all the following phylogenetic and expression analyses.

Species	Abbreviation	Group	Genome	Chromosome No.
*Arabidopsis thaliana*	Ath	eudicots	150 Mb	5
*Arabidopsis lyrata*	Aly	eudicots	206.7 Mb	8
*Brassica rapa*	Bra	eudicots	283.8 Mb	10
*Prunus persica*	Ppe	eudicots	224.6 Mb	8
*Glycine max*	Gma	eudicots	950 Mb	20
*Carica papaya*	Cpa	eudicots	372 Mb	9
*Theobroma cacao*	Tca	eudicots	326.9 Mb	10
*Nelumbo nucifera*	Nnu	eudicots	804 Mb	8
*Vitis cinifera*	Vvi	eudicots	487 Mb	19
*Populus trichocarpa*	Ptr	eudicots	485 Mb	19
*Solanum lycopersicum*	Sly	eudicots	900 Mb	12
*Zea mays*	Zma	monocots	2.3 Gb	10
*Oryza sativa*	Osa	monocots	372 Mb	12
*Musa acuminata*	Mac	monocots	472 Mb	8
*Amborella trichopoda*	Atr	basal angiosperms	706 Mb	13
*Pinus abies*	Pab	gymnosperms	19.6 Gb	12
*Pinus lambertiana*	Pil	gymnosperms	31 Gb	12
*Pinus taeda*	Pta	gymnosperms	20.15 Gb	12
*Physcomitrella patens*	Ppa	moss	473 Mb	27
*Selaginella moellendorffii*	Smo	lycophytes	212.5 Mb	27

**Table 2:** Reference genes used in the study. The table summarizes information on the protein localisation in the plant and predicted protein domains (InterProScan).

Gene Name	Gene ID	Protein Length	Localisation	Flavin- Dependent Monooxygenase Domain	Tryptophan Amino- Transferase Domain	Predicted TMD
YUC1	AT4G32540	414	shoot + cytosolic	yes	no	No
YUC2	AT4G13260	415	shoot + cytosolic	yes	no	No
YUC3	AT1G04610	437	root + cytosolic	yes	no	Yes
YUC4	AT5G11320	410	shoot + cytosolic	yes	no	No
YUC5	AT5G43890	424	root + ER	yes	no	Yes
YUC6	AT5G25620	426	shoot + cytosolic	yes	no	No
YUC7	AT2G33230	431	root and ER	yes	no	No
YUC8	AT4G28720	426	root + ER	yes	no	Yes
YUC9	AT1G04180	421	root + ER	yes	no	Yes
YUC10	AT1G48910	383	flower + cytosolic	yes	no	No
YUC11	AT1G21430	391	flower + cytosolic	yes	no	No
TAA1	AT1G70560	391	whole plant cytosolic	no	yes	No
TAR1	AT1G23320	388	silique + cytosolic	no	yes	No
TAR2	AT4G24670	440	whole plant and ER	no	yes	Yes

**Table 3:** Sequence Read Archives (SRA) files used for RNA sequencing analysis. All SRA files were downloaded from http://www.ncbi.nlm.nih.gov/geo/browse/.

Tissue	SRA File Number for RNAseq Analysis
Root meristematic zone	SRR1740401
	SRR1740402
	SRR1740403
Primary root	SRR1042766
	SRR1042767
Cotyledon	SRR1292207
	SRR605657
	SRR605658
Seedling 14 dag	SRR346552
	SRR346553
Adult Leaves	SRR1159821
	SRR1159827
	SRR1159831
	SRR1159837
Flower	SRR656217
Silique	SRR656218
Flower bud	SRR800753
	SRR800754
	SRR314815

## Supplementary Materials

Supplementary materials can be found at www.mdpi.com/1422-0067/18/8/1791/s1.
