# Supplementary material for: Bioinformatics Analysis of Phylogeny and Transcription of TAA/YUC Auxin Biosynthetic Genes
Source: Int J Mol Sci. 2017 Aug 18;18(8):1791. doi: 10.3390/ijms18081791 (PMC5578179; doi:10.3390/ijms18081791)
Supplement: Supplementary file 1 [file ijms-18-01791-s001.zip › Figure S1,2.pdf]

Supplementary Figures:

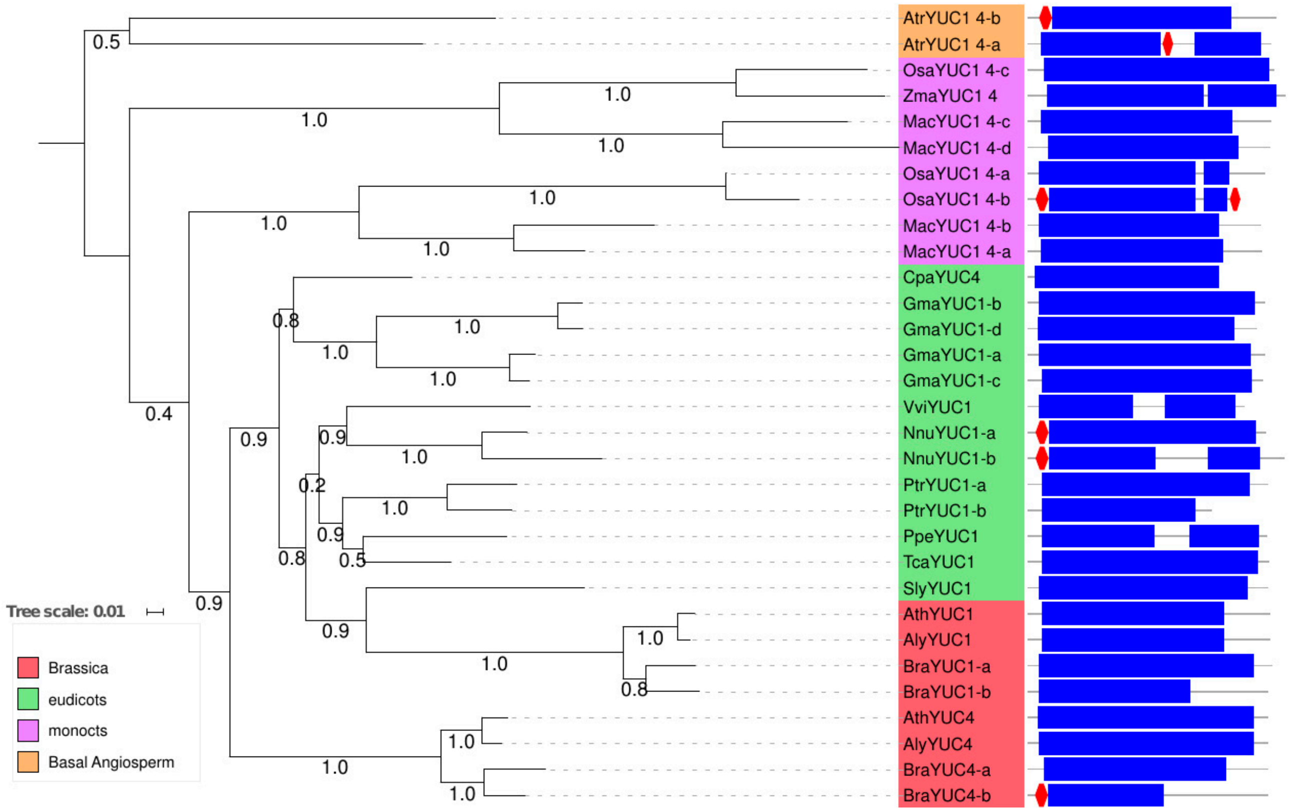

Figure S1. Phylogenetic tree of YUC1 and YUC4 homologues proteins. Fast-Tree using the maximum likelihood method [16] was used to create the phylogenetic tree for YUC1 and YUC4 protein homologues constructed from MUSCLE alignment [18]. Bootstrap values are indicated on the branches of the phylogenetic tree. Gene labels are constructed with the three letters from the species name (Table 1) and the gene name of the *A. thaliana* homologue (Table S1). The color of the label shows the lineage of the plant. The corresponding protein domains (right side) are obtained using InterProScan [15], red hexagons represent the TMDs in the protein if applicable and blue rectangles indicate the flavin-dependent monooxygenase domain typical for YUC proteins.

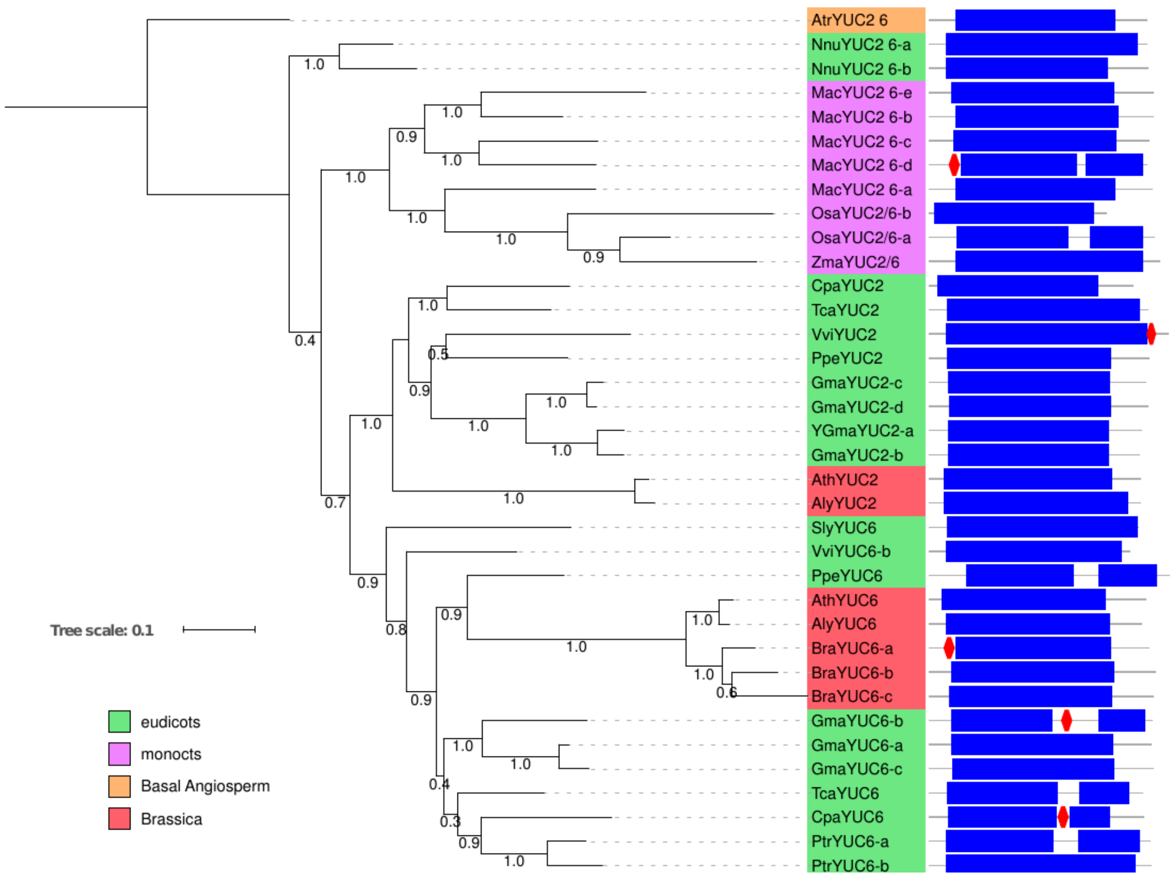

Figure S2. Phylogenetic tree of YUC2 and YUC6 homologue proteins. Fast-Tree using the maximum likelihood method [16] was used to create the phylogenetic tree for YUC2 and YUC6 protein homologues constructed from MUSCLE alignment [18]. Bootstrap values are indicated on the branches of the phylogenetic tree. Gene labels are constructed with the three letters from the species name (Table 1) and the gene name of the *A. thaliana* homologue (Table S1). The color of the label shows the lineage of the plant. The corresponding protein domains (right side) are obtained using InterProScan [15], red hexagons represent the TMDs in the protein if applicable and blue rectangles indicate the flavin-dependent monooxygenase domain typical for YUC proteins.
